# Recurrent Spontaneous Rupture of the Urinary Bladder in a Patient With Human T-lymphotropic Virus Type 1-Associated Myelopathy: A Case Report and Literature Review

**DOI:** 10.5812/numonthly.11764

**Published:** 2014-01-20

**Authors:** Behzad Feizzadeh Kerigh, Reza Boostani, Alireza Ghoreifi

**Affiliations:** 1 Endoscopic and Minimally Invasive Surgery Research Center, Mashhad University of Medical Sciences, Mashhad, IR Iran; 2 Kidney Transplantation Complications Research Center, Faculty of Medicine, Ghaem Medical Hospital, Mashhad University of Medical Sciences, Mashhad, IR Iran

**Keywords:** Urinary Bladder, Myelopathy, Human T-lymphotropic virus 1, Rupture, Recurrent

## Abstract

Recurrent spontaneous rupture of the urinary bladder has rarely been reported in English articles. This condition may be difficult to diagnosis before a laparotomy due to acute peritonitis. Herein we describe a case of recurrent spontaneous rupture of the bladder in a 39-year-old woman with human T-lymphotropic virus type 1 (HTLV-1) -associated myelopathy/topical spastic paraparesis (HAM/TSP).

## 1. Introduction

Recurrent rupture of the urinary bladder is a rare condition, it is usually the result of an underlying disease which may be difficult to diagnose, and the diagnosis is almost always confirmed after a laparotomy (1). In the evaluation of patients with suspected spontaneous bladder rupture, a urodynamic study should also be included (2).

Idiopathic bladder rupture in some patients may be due to a neurogenic bladder dysfunction, which has not been discovered in a clinical and paraclinical evaluation (1). An underlying pathology that weakens the bladder wall is often present, but it either cannot be diagnosed easily or it is missed during the initial evaluation (3). One of the rare underlying diseases that can exhibit neurogenic bladder dysfunction is human T-lymphotropic virus type 1 (HTLV-1) associated myelopathy. Different patterns of urodynamics in these patients are seen according to the course of the disease.

We present a case of recurrent rupture of the urinary bladder in a patient with HTLV-1–associated myelopathy which has not previously been reported in the literature.

## 2. Case Report

A 39-year-old woman presented with a sudden onset of generalized abdominal pain and decreased urine volume. No other lower urinary tract symptoms including; dysuria, or hematuria, were present. In her past medical history there was a history of multiple laparotomies due to different etiologies, including; appendicitis, cesarean section, PID peritonitis and two episodes of spontaneous bladder rupture. The bladder ruptures had occurred two and five years ago, although the initial evaluation did not reveal any etiology and she had not received any special treatment. Her medical records showed that the bladder ruptures had occurred at the dome of the bladder, and it had been repaired with absorbable sutures. She also complained of gradually progressing voiding problems, such as: dysuria, hesitancy, straining to urinate and a sensation of incomplete emptying, for a prior period of about 20 years. This had eventually resulted in an inability to void and she had been using CIC (clean intermittent catheterization) for bladder emptying since this time. No other associated risk factors such as trauma, malignancy or radiotherapy were found in her past medical history. However, she did have a suspicious medical history with a positive HTLV-1 test, without any treatment or follow-up.

**Figure 1.**
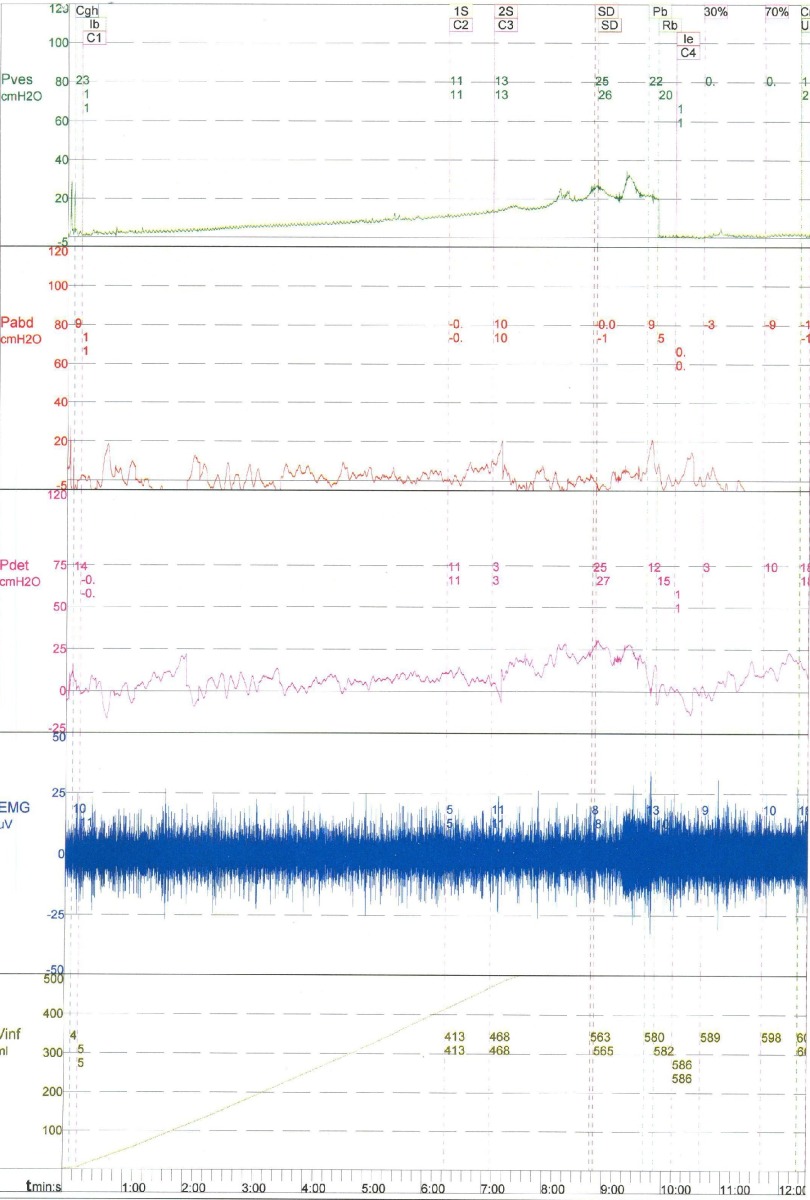
Urodynamic Study Shows Detrusor Hyposensitivity, Hypoactivity and Detrusor Sphincter Dyssynergia (DSD)

On physical examination, there was generalized abdominal tenderness and guarding with positive rebound tenderness. All of the initial blood tests (CBC, BUN, Cr, Na, and K) were normal. A Foley catheter was inserted, but only a small amount of urine was detected. On the basis of her symptoms, the patient underwent an urgent sonography, which showed free fluid in the abdomen, but it did not reveal any hydronephrosis or other urinary pathologies. Based on the clinical and radiological findings, the patient underwent a laparotomy which showed an intraperitoneal rupture of the urinary bladder dome. The site of the perforation was sutured in two layers. The patient remained asymptomatic after the operation. During her follow-up, a cystography study was carried out which showed no pathology, and a subsequent cystoscopy also revealed no pathology. A bladder biopsy was conducted and this showed chronic cystitis. A detailed neurologic examination was performed by a neurologist, which revealed spastic paraparesis. On the basis of the patient’s past medical history and the neurologic examination, a quantitative real time PCR assay was carried out to measure the proviral load of HTLV-1 in her peripheral mononuclear blood cells (PMBCs). It showed a proviral load of 409 copies in 104 PMBCs indicating that 4% of the PMBCs were infected. Therefore, according to her neurologic examination, this lab data was suggestive of HAM/TSP. Further evaluation was done using urodynamic studies, which revealed detrusor hyposensitivity and hypoactivity, and also detrusor sphincter dyssynergia (DSD) (Figure 1). In a cystometry: the first sensation, first desire to void and strong desire to void, were at 408 cc, 462 cc, and 558 cc, respectively. Pdet was achieved at; 11, 13 and 25 cm H2O, respectively. Now the patient is undergoing treatment for HTLV-1 as well as continuing with anticholinergic and CIC, as a result she has remained asymptomatic after her surgery till now.

## 3. Discussion

Spontaneous bladder rupture is a rare event with an incidence of 1:126 000. A total of 79% of all reported cases were in men and the overall mortality was 47% (3). Different risk factors associated with spontaneous perforation of the bladder have been reported in the literature. These risk factors include: blunt trauma to the lower abdomen, carcinoma of the pelvic organs, radiation therapy of pelvic tumors, large ureterovesical stones, bladder irrigation, catheterization of the bladder, atonic bladder, and alcohol intake (4). Some of the reported idiopathic ruptures may have been caused by neurogenic bladder dysfunction, which were not recognized in the initial evaluations (1). There are two different types of bladder rupture; intraperitoneal and extraperitoneal. There is no recognized correlation between the site of the rupture and its cause, but a rupture of the dome, anterior wall and posterior wall are usually seen in; alcohol intake, postpartum women, and patients who have received radiation therapy, respectively (3).

A diagnosis of urinary bladder rupture is difficult to make, and it requires a high index of suspicion. The most common presentation is; diffuse abdominal pain and lower abdominal tenderness, which is often seen due to chemical peritonitis. Confirmation of the diagnosis is almost always done with a laparotomy. Treatment and repair of the perforation is usually performed during a laparotomy, but some patients, especially those with a history of radiation, may be managed conservatively using antibiotic therapy and prolonged bladder drainage (3).

HTLV-1 is a retrovirus that infects about 10 to 20 million people worldwide. Southern Japan, the Caribbean, Central and South Africa, South America and the north east of Iran, are the endemic foci of this virus (5). In 1989, the World Health Organization designated the term HAM/TSP (HTLV-1-associated myelopathy/topical spastic paraparesis) for the neurological syndrome associated with this virus. HAM/TSP is a chronic and slowly progressive inflammatory myelopathy that may lead to severe neurological disability (6). This syndrome is seen in less than 5% of infected individuals (7). Up to 90% of HAM/TSP patients have urologic manifestations (5). Storage symptoms such as; urgency, nocturia, and urge incontinence, are more frequent in the early phases of bladder involvement, but in later phases, bladder-sphincter dyssynergia may be seen, which leads to urinary voiding symptoms such as; dysuria, hesitancy, straining, and a sensation of incomplete voiding, making intermittent bladder catheterization necessary (8).

Urodynamic testing includes the assessment of both detrusor and external sphincter functions. Patients with moderate to severe incontinence, those suspected of having a neurological disease, and those with urge incontinence, as well as patients with obstructive problems, may need urodynamic evaluation for diagnosis and management (5). Castro et al. found prominent urodynamic abnormalities in HTLV-I carriers, which suggest an early compromise of the urinary tract. They also stated that HAM/TSP patients who present with urodynamic findings pose a potential risk to the upper urinary tract (dyssynergia). In their study, detrusor overactivity was the most frequent urodynamic finding in the studied sample and probably, the main cause of the urinary symptoms (5). Furthermore, Lima et al. in their study stated that 80.76% of the TSP/HAM patients showed a hyperreflexic bladder. In total, 34.16% patients had DSD and 82.6% of this group had abnormal uroflow tests. In their study only 3.84% of patients showed a hyporeflexic bladder (9). On the other hand, in a study carried out in Japan by Mori et al., detrusor hypoactivity was the principal finding in the urodynamic evaluations (10).

In the present case, detrusor hyposensitivity and hypoactivity, along with DSD in the urodynamic evaluation, were diagnosed. It appears that rupture of the urinary bladder was caused mainly by bladder overdistention and thinning of the dome wall, secondary to a large volume of urine. Therefore, treatment of the underlying disease and careful regulation of CIC were recommended in order to avoid similar episodes of bladder rupture.
